# Vibroacoustic Impact on the Architectonic Heritage When Using Replicas of 16th Century Weapons

**DOI:** 10.3390/s17081871

**Published:** 2017-08-14

**Authors:** Angel Tomas Lloret, Sandra Sendra, Jaime Lloret, Romina del Rey, Miguel Louis Cereceda

**Affiliations:** 1 Construcciones Arquitectonicas, Universidad de Alicante, Carretera San Vicente del Raspeig s/n, San Vicente del Raspeig 03690, Spain; atllm@hotmail.com (A.T.L.); miguel.louis@ua.es (M.L.C.); 2 Signal Theory, Telematics and Communications Department (TSTC), Universidad de Granada, C/Periodista Daniel Saucedo Aranda s/n, Granada 18071, Spain; ssendra@ugr.es; 3 Integrated Management Coastal Research Institute, Universitat Politècnica de València, C/Paranimf, n° 1, Grao de Gandia 46730, Spain; 4 Centre for Physics Technologies, Universitat Politècnica de València, Camino Vera, s/n., Valencia 46022, Spain; roderey@fis.upv.es

**Keywords:** acoustic impact, structural movements, architectonic heritage, pyrotechnics, vibration, harquebus, blunderbuss, sensors

## Abstract

The recreation of historical battles next to old buildings, walls, churches, fortifications or historical facades belonging to the historical heritage of a city, has always been a source of controversy and discussion. In the absence of a clear legislation about how these buildings can be affected by the use of blunderbusses and pyrotechnics, it is necessary to carry out practical experiments to test the effect of these celebrations on these buildings. For this reason, this paper presents a set of practical experiments where the vibroacoustic effect of using weapons such as blunderbusses and harquebuses is analyzed. To gather these measurements, we have used several sound level meters and 3-axis accelerometers placed on the facade of an old building. The tests have been carried out at the Moors and Christians festival of Villajoyosa (Spain) which is internationally famous for this festival. In order to carry out the tests, six harquebusiers shot their firearms and the sensors placed along the facade of the building at different height collected the data. The results of these devices allow us to study the vibroacoustic impact on the facade depending on the height.

## 1. Introduction

The Corsican battles along the eastern coast of Spain, in the Mediterranean Sea, between the XIV and XVIII centuries, have left a trail of fortifications and watchtowers built for defense purposes. Nowadays, many of these buildings are considered part of the national historical heritage.

Many European countries (especially the ones that border the Mediterranean Sea) celebrate numerous festivals. Such celebrations often recreate battles and events that occurred centuries ago, and in these festivals, large amounts of gunpowder are used in the form of fireworks, firecrackers, and shots from weapons. These elements produce large rumblings, accompanied by a subsequent blast. In addition, these recreations are usually carried out in the old quarters of the towns taking advantage of the beauty of the scenery. The preservation of this heritage represents an interesting tourist attraction which implies an economic benefit for that country [1]. In addition, the use of fireworks and pyrotechnics are not unique to these celebrations. They are also widely used as a means of signaling and beaconing in the livestock industry, agriculture, fisheries, and mining, among others.

To monitor these activities we need to use sensor devices capable of perceiving a physical stimulus from the environment and translating it into a signal or a measurable parameter. In most cases, these sensors need additional electronics to show the measurements in an understandable way [2]. 

There are many applications where acoustic waves and sensor deployments provide great benefits [3]. Regarding to the use of acoustic waves, one of the most used applications is the detection of gas and water leaks [4,5]. Fluid leaks that flow through pipes often generate small vibrations [6] and even small acoustic signals. By capturing these parameters, it is possible to detect such leaks [7].

One the other hand, buildings are sometimes subjected to hazards such as earthquakes and strong winds, fire or vandalism, among others. In order to mitigate, or at least to monitor these facts, researchers generally use sensors and sensor networks. These architectures can measure acceleration, displacement, strain, temperature, smoke, acoustic pressure, etc. Buildings may face many risks that should be monitored, such as aging of structural performance, fatigue, damage, gas leaks, fires, etc. From the results of this monitoring, researchers or owners can take appropriate actions such as structural control, maintenance, evacuation guidance, warning, alarm, firefighting, and rescue or security measures, among others [8].

There are very few papers published regarding the use of sensors for monitoring and protecting our architectural heritage, although one field where sensors are often used is in the monitoring of artworks [9] and frescoes [10].

In this paper, we present the design and development of a series of experiments to study the vibroacoustic impact that the use of blunderbusses and harquebuses can cause on the historical heritage. The tests should help us to determine if these old buildings would be affected by the effect of pyrotechnics. The acoustic impact is understood as the magnitude of the mechanical wave that impacts the facade. Depending on the amplitude of this mechanic wave, which will be measured by using sound level meters, the facade may suffer some movements that can be perceived as a vibration by accelerometer sensors. With these two parameters, we will evaluate the vibroacoustic impact over the facade as a function of the height.

In order to carry out this work, we selected an old building located in Villajoyosa (Province of Alicante, Spain) which is famous for its Moors and Christians festival. Several sound level meters and vibration sensors were installed on the facade. The noise was generated using the synchronized shots of six harquebusiers. The firearms used are reproductions of actual weapons used in these ancient battles. From the results, we have drawn vibroacoustic impact maps of the facade. The results showed that the impact over the facade could have serious implications such as the detachment of elements of the facade that could both harm people and generate irreparable damage on these monuments that we want to protect.

The rest of this paper is structured as follows: Section 2 presents some papers where the authors carried out studies on the effects of acoustic impacts on building facades. Section 3 describes the scenario where the tests have been carried out and the tools and materials used during the tests. The experiments carried out and the measurement results are discussed in Section 4. Finally, Section 5 presents the conclusions and suggestions for future work.

## 2. Related Work

There are several studies related to the analysis of acoustic impacts on buildings. However, they are more focused on the study of the insulating characteristics of housing to ensure the welfare of its inhabitants. This is the case of a study presented by Park et al. [11] where the authors examined how residents in apartment buildings perceive and react to impact sounds coming from their upstairs neighbors’ dwellings. The authors described the noise annoyance and non-acoustic factors. In addition, the authors stated that the noise sensitivity of a person had a direct impact on the perceived disturbance and an indirect impact on its annoyance. Finally, the results showed that the annoyance generated by the impact noise on the floor was associated with self-reported health complaints. 

An interesting example related with the surveying of ancient buildings was presented by Fregonese et al. [12]. This work describes some techniques for surveying ancient buildings. In this case, the authors used a Terrestrial Laser Scanner (TLS) in combination with traditional topographic techniques such as geometric leveling and topographic networks for 3D control to survey and study the Palazzo del Capitano in Mantua (Province of Mantua, Italy). The main aim of this study was the detection of surface displacements in buildings. For the case of this specific building, the authors analyzed images taken over time to see if any part of the structure of this palace had undergone some structural movement. The authors conclude that nowadays, the known techniques still present serious limitations for monitoring ancient buildings. Costanzo et al. [13] used similar techniques for analyzing the St. Augustine Monumental Compound, located in the historical center of Cosenza (Calabria, Italy).

Cuadra et al. [14] presented a study to estimate the dynamic characteristics of Inca stone structures due to microtremors that generate very small displacements. The authors assumed that stone structures present an elastic behavior capable to withstanding small strain conditions. From their results, the authors proposed a new method to evaluate the seismic behavior of these constructions and consequently the seismic vulnerability of these structures.

Ceriotti et al. [10] described the development of a wireless sensor network on Torre Aquila, a medieval tower located in Trento (Province of Trento, Italy). The system was based on a set of motes called TMotes, 3MATE! environmental nodes and fiber optic sensors stretching the length of the tower to measure its deformation. The tests were performed during four months. The authors concluded that WSN-based monitoring systems are effective tools to assess a tower’s stability, and deliver the needed data to monitor this kind of structure.

As we can see in these previous works, there is significant interest in monitoring and analyzing the status of our cultural heritage, but this section tries to be more focused on previous works where the authors analyzed the effect of sound pressure on buildings. 

Firstly, Karatzetzou [15] developed three different applications of ambient noise measurement methods to measure the foundation-soil stiffness in terms of shear-wave velocity, the fundamental frequency of the structure in its present state, the mechanical properties and the distribution and intensity of damage to masonry walls. The authors analyzed the ambient vibrations measurements on three monuments in the medieval city of Rhodes (Island of Rhodes, Greece) and the surrounding soil. From the results, the authors identified the dynamic characteristics of these structures and the foundation-soil where these structures were located.

We should also highlight that it is easy to find studies [16,17] where authors analyzed facade sound insulation and material response as a function of the working frequency and the source that generated the noise. Yu and Kang [18] also presented a study to analyze the differences in environmental impact between different architectural acoustic materials in residential buildings. The study was performed on five houses with different infrastructure, i.e., the authors used a bungalow, detached, semi-detached, and terraced houses and apartments to carry out their tests. For each building, the authors compared several common wall materials with similar sound transmission losses and three different glazing ratios. The results showed the importance of considering the environmental sustainability of acoustic materials when planning to build a house.

There are very few works where the practical study also includes measurements regarding to vibrations of structural movements. This is the case of Klos [19] who performed a study to analyze the vibroacoustic response of buildings due to sonic boom exposure. To do this, the authors used 112 transducers installed in three bedrooms of a house. Accelerometers were attached to the walls, windows, and ceilings of these three rooms and microphones were placed at random locations in each room and in the surroundings of the house to characterize the resulting noise indoors and the effect of diffraction of the boom around the house. The tests included shaker excitation of the walls and windows of the house and reverberation time measurements, among others, inside and outside the house at various locations.

Finally, regarding the use of weapons, one of the first works related to the acoustic characteristics of harquebuses and blunderbusses was performed by Marco Sanjuán in 1996 [20]. In this work, the author performed a set of measurements of the sound levels of different types of commercial gunpowders. This study does not cover the three types of explosive used for the different models and sizes of historical weapon reproductions, but the obtained values are an interesting indicator to start with our measurements of sound pressure over a facade.

As far as we know, there is no study that relates the use of weapons during re-enactments of these ancient battles and the effect of these on the cultural heritage. We think it is important to maintain these celebrations, their reproductions, and memory as faithful as possible to the actual facts, however, we also believe it is important to preserve the monumental remains and our historical heritage. It is, therefore, important to characterize how historical buildings may be affected by the use of this type of fireworks.

## 3. Scenario and Material Description

This section shows the scenario where the tests have been carried out and the tools and materials used during the different tests.

### 3.1. Scenario

**Figure 1:**
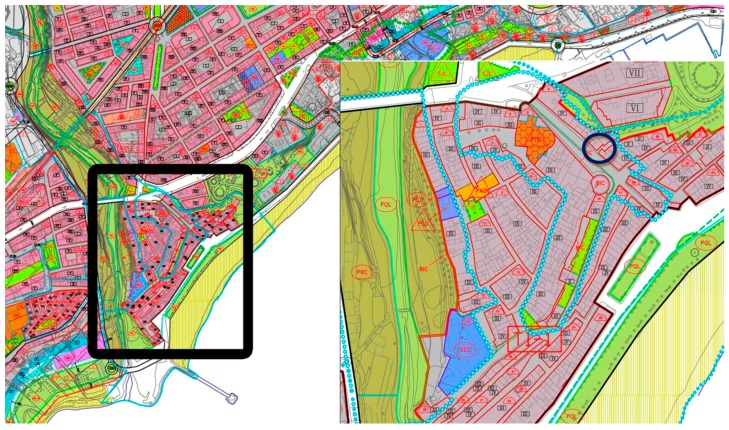
Section of the urban plan of Villajoyosa (Alicante, Spain).

To perform these tests, we have chosen an uninhabited house of ancient construction since we cannot expose old architectural elements to the stress of these experiments. This property is located in a pedestrian street in the historic city center. In the same area, we can find many historical sights of the city such as the defensive walls built between 1551 and 1565 to repel Berber attacks. Figure 1 shows a section of the urban plan of Villajoyosa (Alicante, Spain) and the area where the tests have been performed.

The building is a block placed on the historical center of Villajoyosa, declared as Heritage of Cultural Interest (in Spanish *Bien de Interés Cultural-BIC*), a denomination given by the Spanish Government for not only material heritage, like monuments or movable works of art, but also intangible cultural heritage). The building presents a rectangular base and it is placed between other buildings. The foundation base around the perimeter is scarce or nonexistent. The structural support function is performed by load-bearing walls that present higher thickness than normal. These are buried and mainly transmit the loads to the ground by friction. The building has a surface structural system based on load-bearing walls of masonry mortar, as has been observed in various historical documents. Load-bearing walls that form the facades typically have a coating of hydraulic cement and sand mortar, and a traditional plaster cover of lime and sand. The load-bearing walls decrease in thickness as the building height increases. Finally, as a vertical communication element, there is a staircase that connects the different floors. It is built as a brick vault where the stairs are placed.

**Figure 2:**
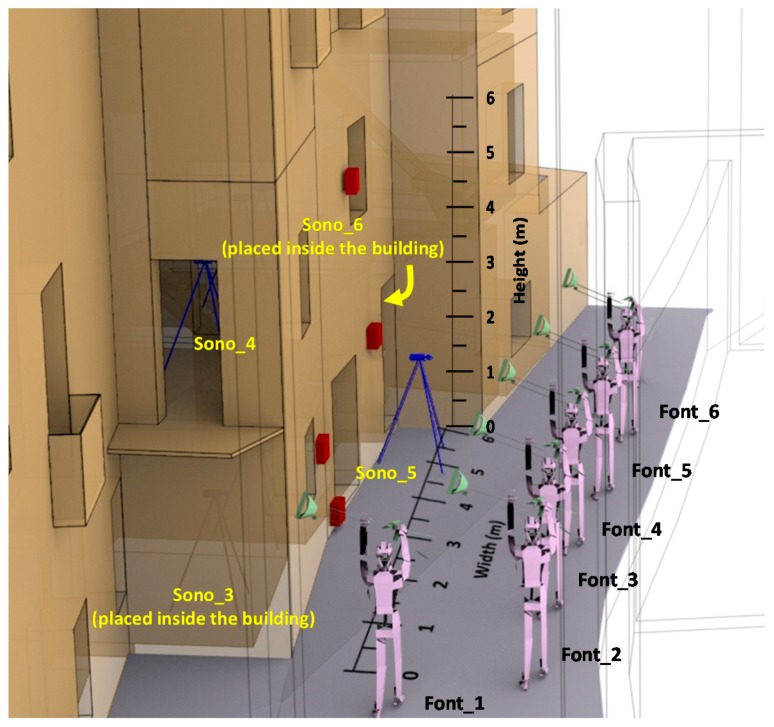
Scenario where measurements have been performed, and the position of harquebusiers and sound level meters.

Figure 2 shows a scale drawing of the scenario where the tests were carried out. This picture also shows the position of the harquebusiers, the positions where the sound level meters have been placed as well as the dimensions of the facade section exposed to our tests. The surface of our facade is 6 m wide and 6 m in height. To perform the tests, six harquebusiers and four sound level meters have been used. Each harquebusier is identified as “Font_X” where X indicates the harquebusier. Sound level meters are identified as “Sono_Y” where Y indicates the device. This nomenclature is used in the rest of paper. 

### 3.2. Material Used to Perform the Tests

This subsection describes the features and sizes of the weapons used and technical data such as the maximum sound pressure levels that these weapons can reach and the physical properties of the black gunpowder used during the tests.

#### 3.2.1. Weapons Used in the Replica of the Real 16th Century Battle

**Figure 3:**
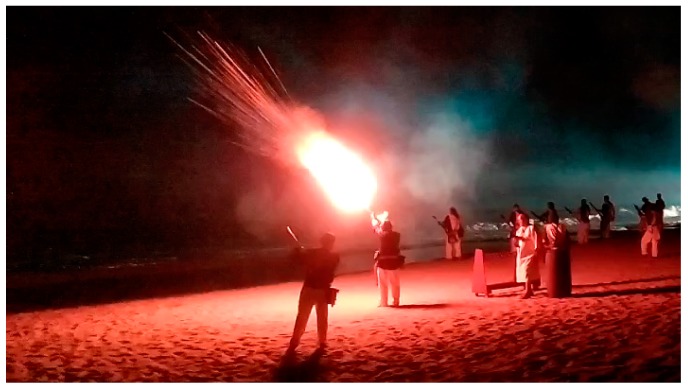
Harquebusier in a replica of battles.

During the replica of these centenarian battles, harquebusiers use firearms without bullets which generate a large flare (See Figure 3). Harquebusiers usually use blunderbusses or harquebuses. Both are firearms with small differences between them:

*Blunderbuss*: These are short muzzle-loading firearms with a large caliber; it is destined to fire shrapnel or small pellets. This kind of firearm is generally used over short distances. The blunderbuss is usually activated by a spark, although there are some models that are activated by percussion or small detonators (pistons). They were often used by bandits and smugglers. The blunderbuss is the successor of the earlier “petrol” used by bandits and Catalan supporters of Christians troops during the 17th century.

*Harquebus*: This is an old firearm with an iron gun barrel and a wooden box. The name “harquebus” is due to the arcuate shape of the mouth that was modified in the blunderbuss to facilitate the addition of gunpowder. The harquebus is a firearm that belongs to the muzzle-loading category, i.e., it is loaded through the muzzle. It is smaller in size than the blunderbuss, with a bore established between 14 and 18 mm. According to its construction and type of chamber, a harquebus can load from 15 g to 50 g of black powder. The fireplace primer and the firing pin driven by the crossbow are placed on the back of the gun barrel. The muzzle of the weapon has a flared look for easy loading.

**Figure 4:**
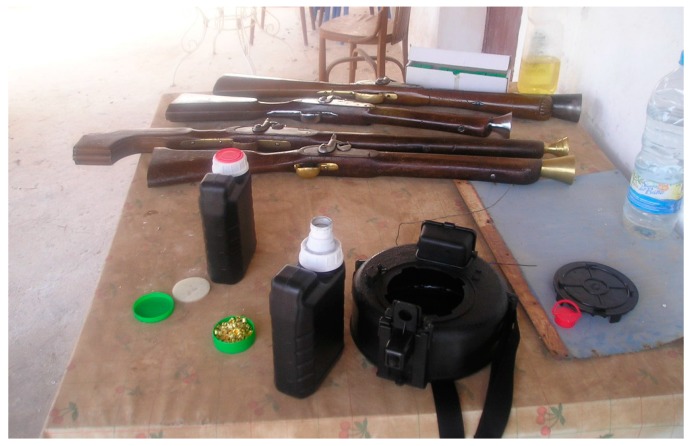
Weapons, explosives detonators and transport container used in the replicas of battles.

Figure 4 shows some weapons, explosive detonators and transport containers used in the battle replicas. 

#### 3.2.2. Industrial Explosive Mixtures Used in the Detonations

Mine gunpowder is not an actual explosive product since its reaction deflagrates instead of detonating. The feature of this product is mainly its large volume of combustion gases. It is a ternary mixture of sulfur, potassium nitrate and charcoal. Another important feature is its high combustion temperature. Its reaction rate is low compared with the rates given for the denotation of explosives. Depending on the caliber, the rate can reach speeds to 500 m/s.

Black gun powder is quite sensitive, so it is necessary to maintain strict safety measures while handling it. The mine powders are supplied in granulated form. At the end of the manufacturing process, graphite is added because its conductive nature prevents the formation of electrostatic charges. It also provides a lubricating action so that good fluidity is achieved. 

The detonation of any explosive always produces a certain proportion of gases that are toxic to the human body. The greater or lesser effect depends on other factors such as the composition of the explosive itself and the amount of oxygen dissolved in it. Table 1 shows the physical properties of black gunpowder used in harquebuses and blunderbusses.

**Figure 5:**
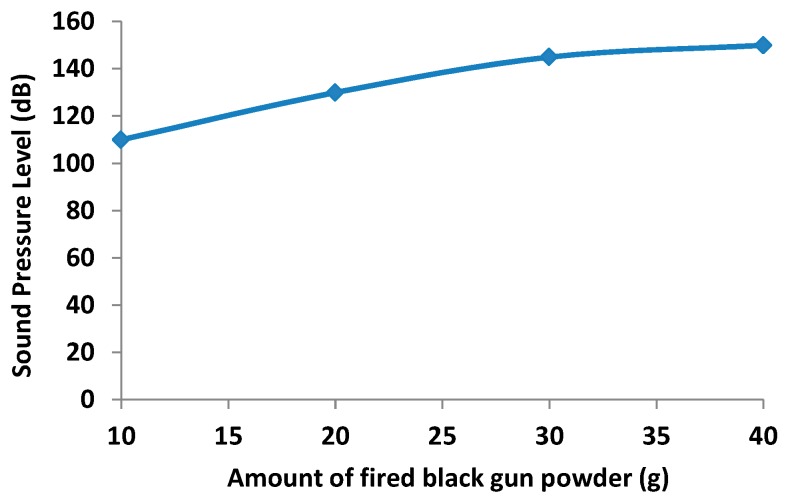
Sound pressure level as a function of black gun powder used.

Black gunpowder is a mixture of potassium nitrate:carbon:sulfur (KNO_3_:C:S) which percentages are 75%:15%:10% (potassium nitrate can be replaced by sodium nitrate). It is usually prepared wet to ensure that the sodium or potassium nitrate particles impregnate the C and S particles. In this way, the mixing process offers better results. Only 50% of black gunpowder becomes hot gases when burned; the rest is burnt as very fine particles. Figure 5 shows the relationship between the black gunpowder used in shooting and the sound level pressure registered at 5 m.

To carry out the tests performed in these experiments, we have used black gunpowder marketed by the company Maxam Outdoors (Madrid, Spain) whose characteristics are [21]:

- Grain size: From 0.35 to 1.19 mm.
- Amount of KN0_3_: 74%.
- Container Size: 1 kg.

### 3.3. Explanations about the Test Bench

**Figure 6:**
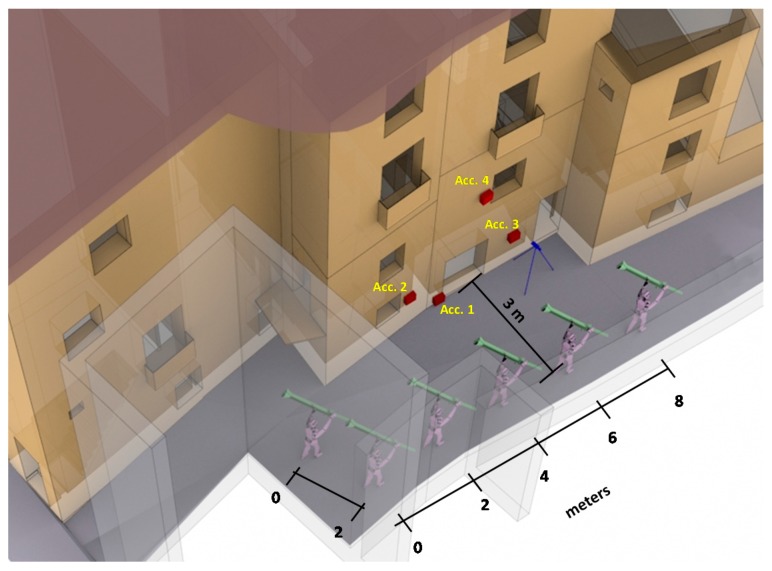
Distribution of harquebusiers and accelerometers.

In order to perform these tests, we have required the presence of six experimented harquebusiers placed as shown in Figure 2. In our case, the impact study is conducted in several rooms of a house that lacks furniture, which may modify the results. The measurements are made at different heights. The six noise sources are located outside the building under study. The distance between harquebusiers is 2 m and the distance between the building and harquebusiers is 3 m (see Figure 6). 

In regard to the location of the sensors, the sound level meters are located within the building located at 2 m from the inner side of the facade, window or access door. With this distance, the reflections over the reflecting surfaces of the building itself do not affect the measurement. Furthermore, because the building does not contain furniture, we can be sure that no additional element will interfere with the measurements results. The vibration sensors take measurements at three axes. They are fixed to the outside part of the facade with bee wax that gives freedom of movement to the mobile parts of accelerometer sensors. Figure 6 also shows the location of the vibration sensors. They are identified as “Acc X” where X indicates the device. Table 2 lists the characteristics of the weapons used in this study.

In the performed shots, we have not used metal projectiles. Therefore, to imitate the sound of a real shot, the amount of explosive used must be increased with amounts of up to 40 g of explosive deflagration inside the weapon. In our case, we have used several historical weapon reproductions the same type (muzzle-loading) with different sizes and dimensions. The amount of explosive has been the same in all cases.

**Figure 7:**
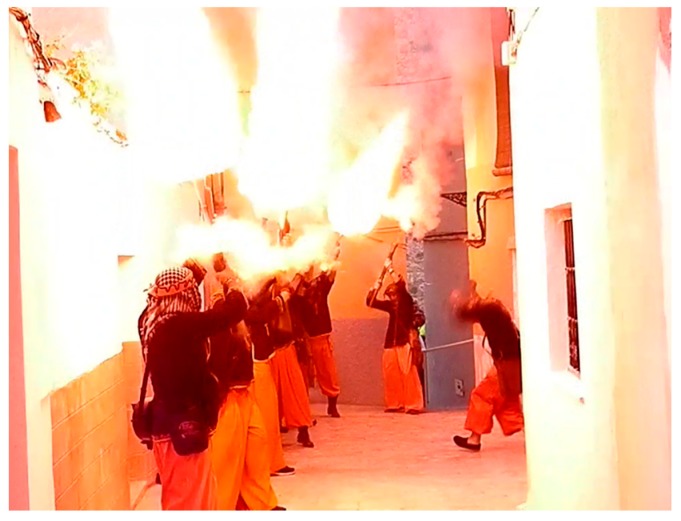
Harquebusiers carrying out a shot where the explosive deflagration is observed.

The position adopted by the harquebusiers is conditioned by security aspects, i.e., the residue of shot and flame should not interfere on other harquebusiers or viewers. The second reason for adopting this position is to keep the weapon in a safe position after the recoil caused by the explosion inside it (see Figure 7). In this position, the weapon must point to the sky at an angle of 45° from the horizontal with the trigger. The gun must be kept over the head of the harquebusier for safety reasons.

**Figure 8:**
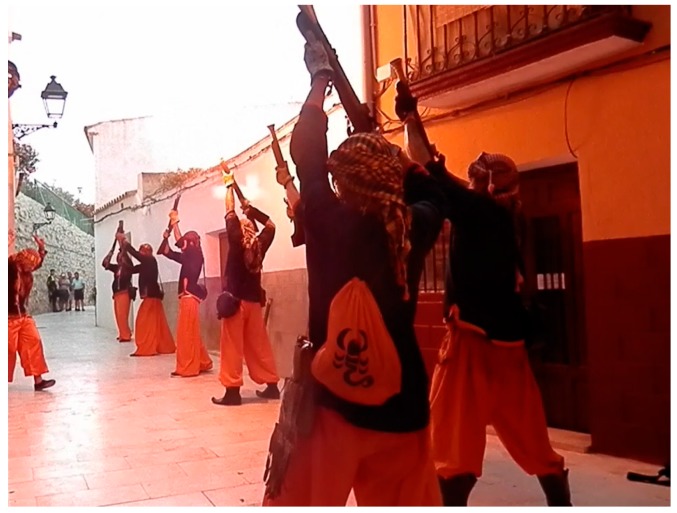
Harquebusiers in the safety position ready to perform a shot.

Finally, the weapon must remain turned outwards on its longitudinal axis to avoid any kind of incident if the deflagration comes back down the barrel (see Figure 8).

**Figure 9:**
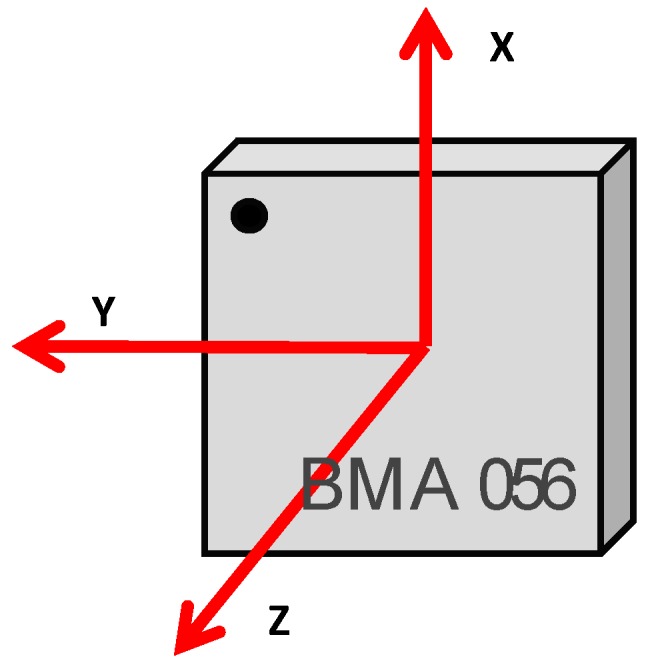
BMA056-3-Axis accelerometers.

The vibration sensors used in our study were Bosch BMA056-3 axis accelerometers [22] (see Figure 9). The encapsulation size is 3 × 3 × 0.95 mm^3^. It is one of most used models for virtual reality and navigation applications, motion tracking, shock and vibration detection in smartphones and mobile devices. This sensor is able to measure magnetic field levels in the range of ± 1000 μT (for X-, Y-axis) and ± 2500 μT (for Z-axis) with a resolution of 0.3 μT. Regarding the accelerometer measurements, the Bosch BMA056 has a resolution of 12 bits and a sensitivity tolerance of ±4%. In addition, this sensor can be programmed in different ranges ±2 g; ±4 g; ±8 g; ±16 g and Zero-g offset of ±80 mg. In order to collect and save the data from acceleration sensors, an ESP8266 node has been used. The acceleration sensors are connected to the node through I^2^C bus. Finally, the sampling rate is 12.5 Hz.

**Figure 10:**
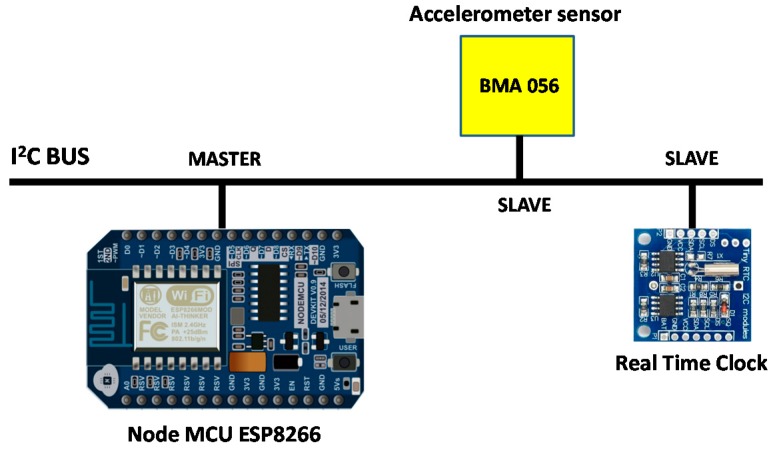
Schema of connections.

In relation to the synchronization of the accelerometer sensors, the sensors are initialized using a real-time clock connected to the ESP8266 node. This device allows us to have a temporal reference to perform the measures. The real-time clock and the acceleration sensor are slave elements within the communication through the I^2^C bus while the ESP8266 node acts as the master. Figure 10 shows a schematic of the connections.

**Figure 11:**
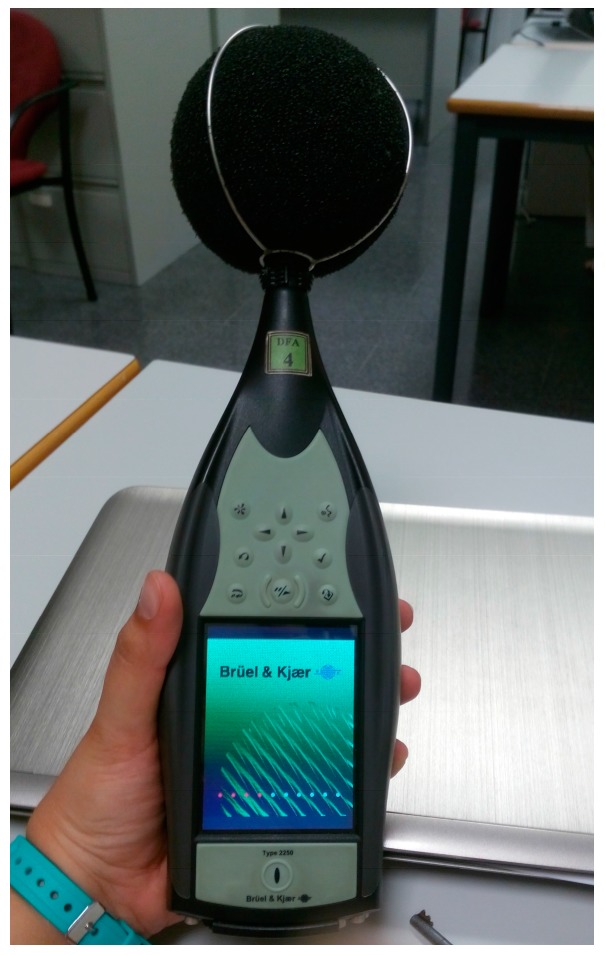
A Bruël & Kjaer Type 2250 sound level meter.

The sound pressure measurements have been performed using professional sound level meters. In these tests, we have selected the Bruël & Kjaer Type 2250 sound level meter [23]. It performs Class 1 measurements according to the international standards. This sound level meter has a wide dynamic range that supports both the most intense noises and those barely perceived above the background noise. Moreover, the Bruël & Kjaer Type 2250 sound level meter is able to measure from the low-frequency option, ranges from 20 kHz down to infrasound for measurement of noise sources suspected of emitting very low frequency noise. The device can perform in 1/1 octave and 1/3 octave bands. Figure 11 shows one of the sound level meters used in the test bench.

Finally, it is important to know the environmental conditions because acoustic waves are highly affected by the temperature. Moreover, very high wind speeds can mask the measurement results. As an important datum, when the speed wind is higher than 5 m/s, the resulting acoustic measurements are not valid [24]. In our case, the following environmental values were registered:

- Temperature: 29.1 °C.
- Relative Humidity: 75.3%.
- Speed wind: 1 m/s.

With these values, we can perform the tests and we can be sure that the registered values really represent the effect of the harquebuses and sound pressure on the facade.

## 4. Tests Performed in the Real Scenario and Measurement Results

This section shows the experiments carried out in the scenario presented in the previous section. We also discuss the measurement results. These results show the acoustic and seismic impact on the building.

### 4.1. Acoustic Impact and Building Movement Results

In order to perform the tests and take measurements of sound pressure levels and vibration recorded at different points of the facade, the harquebusiers made two sets of shots. This subsection shows the values registered by the sound pressure level and vibrations registered at different points of the facade during the two sets of shots. 

#### 4.1.1. Considerations over the Performed Measurements

When measuring the acoustic impact, we should take into account the effect we want to measure. This fact will determine the frequency weighting we should use. Frequency weightings correspond to acoustic sound issues and have a large psychoacoustic component. 

Acoustic analyzers aim to approximate their responses to the human ear. While Sound Pressure Level (SPL) in dB describes the physical phenomenon, the weighted decibel level describes the existing loudness, i.e., the perception of a sound by a human. In our case, we want to measure the physical effect over the facade and therefore we will use linear weighting. Linear weighting is a flat frequency response between 10 Hz and 20 kHz ± 1.5 dB, excluding the microphone’s response. The results directly represent the effect we want to measure. All information concerning this weighting is presented in the international standard IEC 61672: 2013. Our measurements are performed in Z weighting, according to Bruël & Kjaer nomenclature (measured in dB) in the 1/3 octave band [25,26,27].

#### 4.1.2. Values of Sound Pressure Level Registered over the Facade

**Figure 12:**
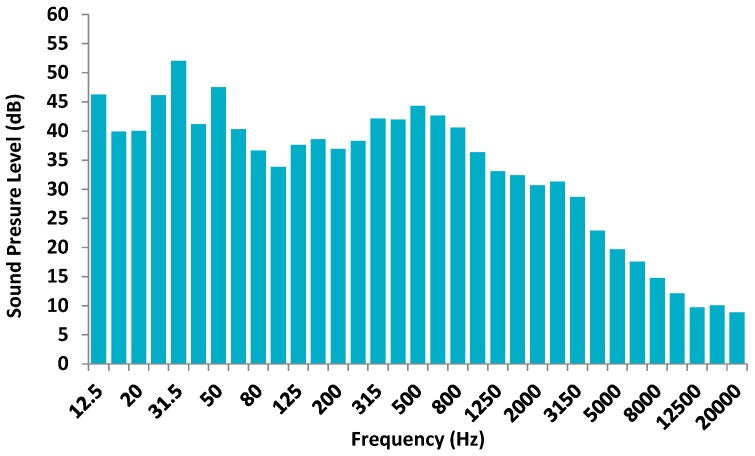
Average background sound pressure (dB).

First we analyze the average sound pressure level received by the facade when there is no activity, i.e., without pyrotechnics or harquebusiers performing shots. Figure 12 shows the average value (calculated from the data from all sound level meters) of the background sound level in dB registered on the facade. 

We can see that maximum value of sound pressure is 52 dB at 31.5 Hz and around 45 dB at 12.5 Hz, 50 Hz and 500 Hz, i.e., the highest values are registered at low frequencies. Low frequencies are the ones which present the biggest impact over the buildings and are the ones which most disturb people.

**Figure 13:**
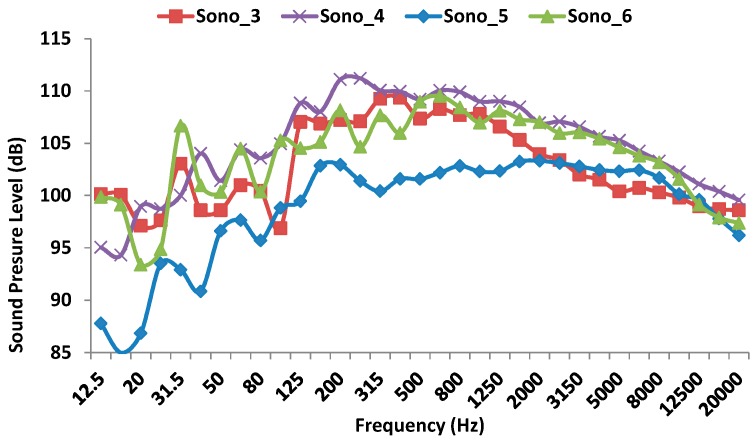
Sound pressure level measured by the four sound level meters during the first shot.

Figure 13 shows the sound pressure level measured by the four sound level meters during the first shot. As we can see, the sound level meter that registers the lowest sound pressure level is the one placed at lowest altitude respect to the floor (103 dB). Moreover, the rest of devices record pressure sound levels around 110 dB. The biggest noise impact is generated in the range of 200–250 Hz.

**Figure 14:**
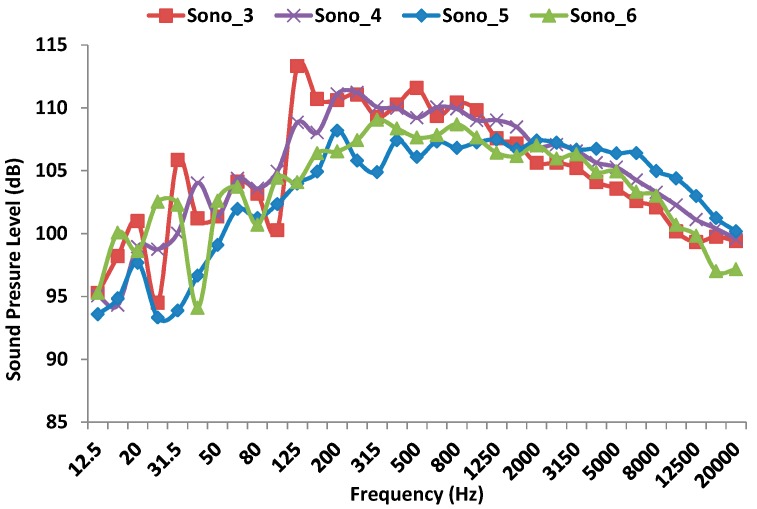
Sound pressure level measured by the four sound level meters during the second shot.

Figure 14 shows the sound pressure level measured by the four sound level meters during the second shot. Again, the sound level meter that registers the lowest sound pressure level is the one placed at lowest height (approximately 1 m) with respect to the floor (108 dB at 200 Hz). This is Sono_5. Moreover, the rest of sound level meters record maximum values around 111–113 dB in the frequency range of 125 to 300 Hz. From this value, the received signal is attenuated. The biggest sound impact is recorded at 125 Hz. by Sono_3 (with a value of 113.31 dB), which is approximately at 1.60 m height.

**Figure 15:**
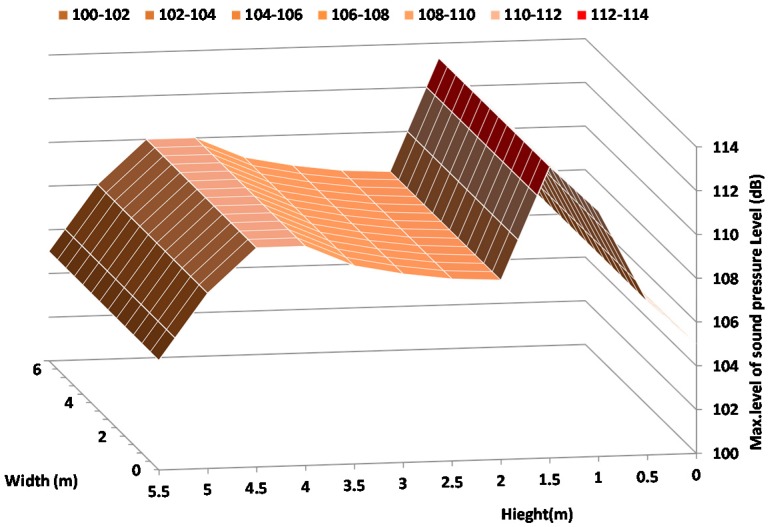
Map of maximum values of sound pressure level registered on the facade in (dB) as a function of the height.

Finally, Figure 15 shows the map of maximum levels of sound pressure on the facade as a function of the height and width of facade. Figure 13 shows the maximum value registered simultaneously in all sound level meters which are placed on the positions specified in Figure 2. As we can see, the highest value is registered at 1.5–2 m (which corresponds to the height of the ground floor) with a value around 113 dB. We can also see that a sound pressure level peak is registered at 4 m height with a value of 109 dB.

**Figure 16:**
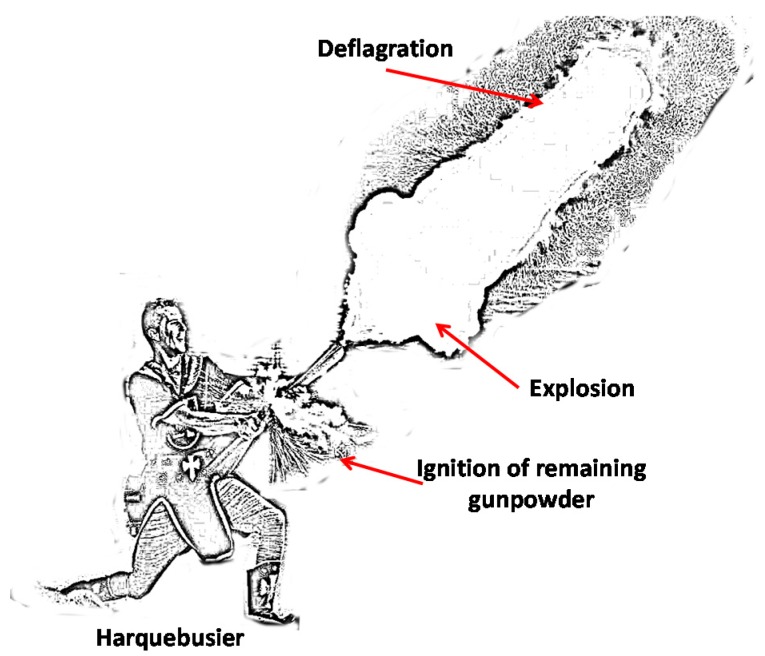
Harquebusier shooting a weapon.

As we can see, there are two areas of greatest sound pressure impact. This is because during a shot, the explosion generated by the weapon has approximately been generated at 1.5–2 m height, while the deflagration can reach several meters (up to 5–6 m) in height and also causes the propagation of sound waves. Figure 16 shows a drawing of a harquebusier shooting a weapon, where it is easy to see the explosion and deflagration.

#### 4.1.3. Values of Vibration Index Registered over the Facade

To start with the measurements of vibration, we should take into account that a facade and a building, in general, only receive vibrations from seismic sources or from industrial machinery called seismic noise, although the human activity and natural phenomena can also generate structural movements.

Usually, when we talk about vibration measurements in buildings, this magnitude is usually represented as a vibration index (Law) in dB [28]. This value is obtained by Equation (1):

(1) $$Vibration\;Index\;\left(\mathrm{dB}\right)=20\cdot \log\frac{acc\;\left(m/S^{2}\right)}{acc_{0}\;\left(m/S^{2}\right)}$$

where, $acc\;\left(m/s^{2}\right)$ is the acceleration measured by the acceleration sensor and $acc_{0}\;\left(m/s^{2}\right)$ is the acceleration reference that depends on the device. In our case, the $acc_{0}=10^{-6}\;m/s^{2}$.

In our case, we are going to measure the effect of shooting on the facade cladding. The most affected elements with the biggest probability of coming off will be those that present a deficient adhesion to the structure. For this reason, the sensors are not embedded in the structure. To fix the sensors, we have used bees wax which is widely used when vibration dosimeters are employed. The sensors are in contact with the elements of structure that could be detached by the effect of the received acoustic waves. This subsection shows the results of the vibrations detected over the facade cladding during the two shots.

To process the data from the 3-axis accelerometers, we have measured the components, i.e., the acceleration registered in X-axis, the acceleration registered in Y-axis and the acceleration registered in Z-axis. After that, these values are combined using Equation (2):

(2) $$a_{Combined}=\sqrt{a_{x}{}^{2}+a_{y}{}^{2}+a_{z}{}^{2}}$$

where, $a_{x}$, $a_{y}$ and $a_{z}$ are the values of acceleration registered in each orthogonal axis, X, Y and Z.

**Figure 17:**
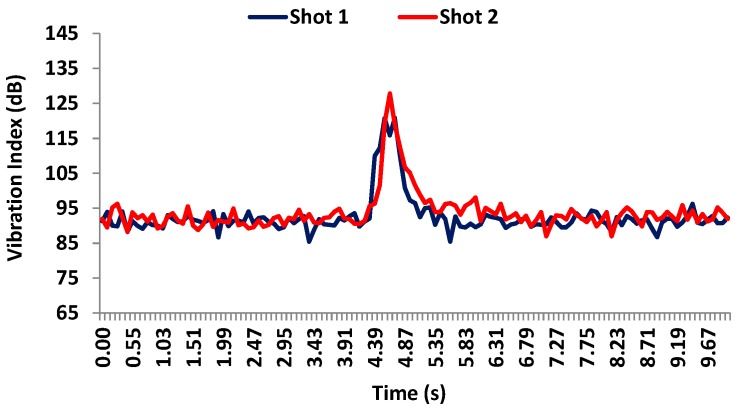
Vibration levels registered by accelerometer 1 on the facade during the shots.

The measurement time for each case is 1 min, but we have selected the time during which the movements has been detected. The measurement results are as follows: firstly, Figure 17 shows the vibration values registered by accelerometer 1 during the shots. We can observe that the maximum amplitude is around 128–130 dB for both cases while the rest of the time, the sensor registers an average value of 93–94 dB as ground vibration. The recorded impulse is narrow which implies very low structural movements. This is because this sensor is placed on the ground floor.

**Figure 18:**
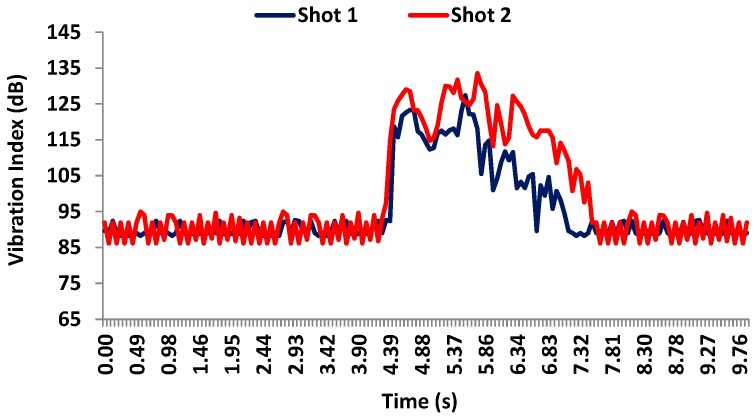
Vibration levels registered by accelerometer 2 on the facade during the shots.

Figure 18 shows the values registered by accelerometer 2 during the both shots. In this case, shot 2 registers a slightly bigger vibration (133 dB) than shot 1 which reaches 125 dB. The ground vibration is around 85 and 90 dB for both shots. The sensor results also show that shot 2 has needed around 3.2 s in order for the wall to totally absorb the vibration. For shot 1, this time is smaller since the maximum registered level has been lower. This sensor is placed at 2.20 m height.

**Figure 19:**
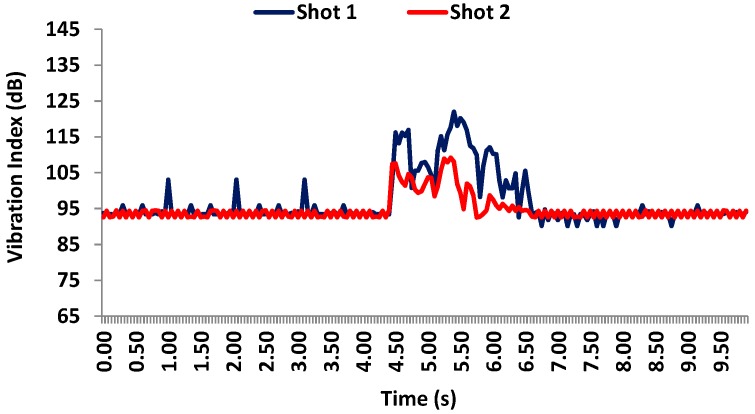
Vibration levels registered by accelerometer 3 on the facade during the shots.

Figure 19 shows the values registered by accelerometer 3 during both shots. In this case, shot 1 registers a bigger vibration (124 dB) than shot 2 which reaches 113 dB. The rest of time the vibration level is around 94 dB for both shots. We can also see that the vibration needs around 3 s to be totally absorbed. This is because this sensor is placed at 1 m height.

**Figure 20:**
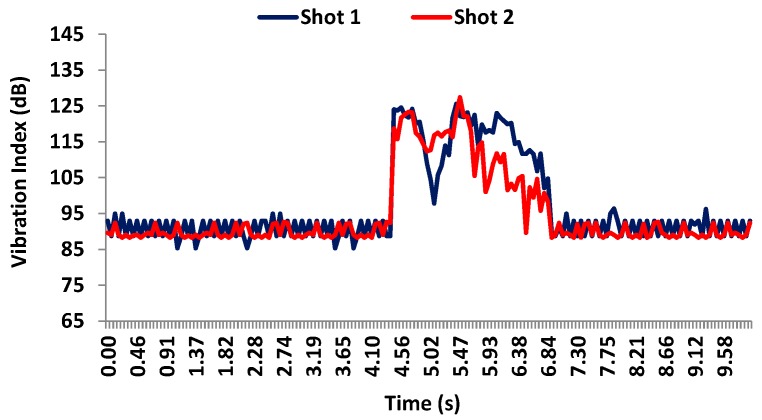
Vibration levels registered by accelerometer 4 on the facade during the shots.

Figure 20 shows the values registered by accelerometer 4 during both shots. Both shots register similar values, i.e., 125 dB). The ground vibration is around 92–93 dB for both shots. Again, the results have shown that the facade needed around 3.2 s in order to totally absorb the vibration. This sensor is placed at 4.5 m height.

**Figure 21:**
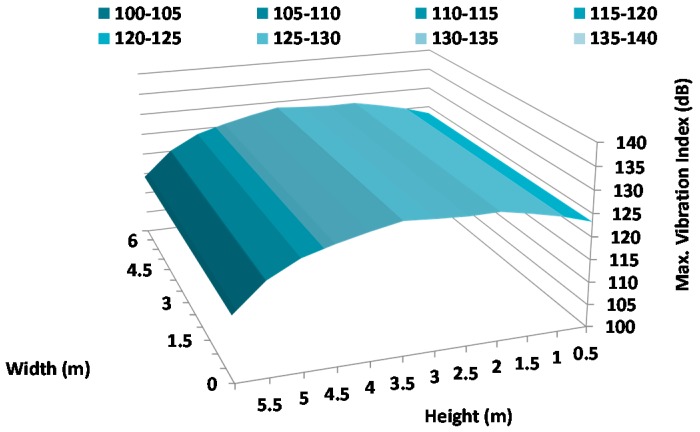
Maximum variation of vibration in the X-Axis.

**Figure 22:**
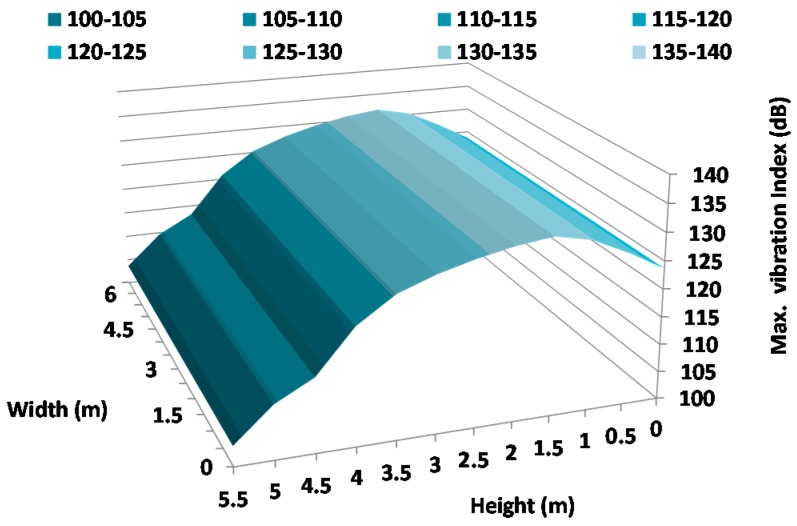
Maximum variation of vibration in the Y-Axis.

**Figure 23:**
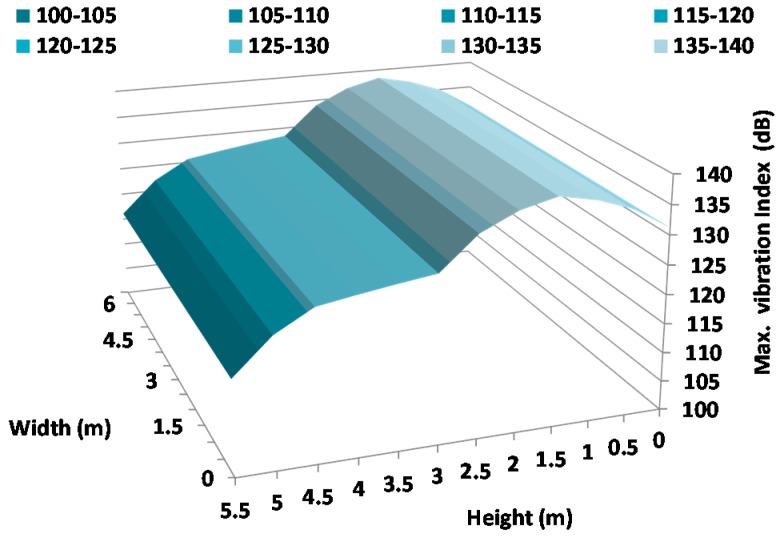
Maximum variation of vibration in the Z-Axis.

Figure 21, Figure 22 and Figure 23 show the vibration maps on the facade for the X-axis, Y-axis, and Z-axis of the sensors, respectively. To make them, we have taken the maximum value of the vibration index registered at the same time at different places on the facade. These places are the sensor positions and because they are placed at different heights, these figures show us the movements as a function of different heights. 

As we can see, the highest vibration index is registered in the Z-axis (Figure 23). In addition, the highest value is registered around 1.5 m with maximum values of 133 dB which corresponds with the height where the harquebuses generate the highest sound pressure level. The axis that registers the lowest vibration is the X-axis, with a maximum value of 128 dB at 3 m height. As we can conclude, the Z-axis registers the biggest vibration index since the sensors register the movements in the transversal sense of the wall which presents a larger freedom of movement.

### 4.2. Discussion

Initially, this study was carried out to find out if there is any risk to people and built heritage in order to ensure the perpetuity of a traditional celebration. In any case, the security of people should prevail over any type of patrimony. For this reason, the human risks derived from this activity in terms of the historical scenario are a result of the possible detachment of objects from the facades onto the people. 

To carry out the test, the most unfavorable acoustic site has been established, i.e., a narrow street with tall buildings, and the most unfavorable situation of the shooters, i.e., a big concentration of harquebusiers with an excess of gunpowder in the weapon.

The purpose of this test was to detect the vibrations caused by the most unfavorable case of these festivals where elements such as the eaves, cornices, carpentry, balconies, and facade cladding can fall off and harm people and these detachments could be irreversible and consequently the historic heritage would remain damaged 

From the results, we can make several observations. Firstly, it is clear that the use of these weapons causes significant movements on the building facade. In regard to the acoustic impact, we note that the highest level is recorded at 1.5 m. The sound pressure levels recorded show some values higher than 113 dB at 160 Hz. However, there is another area where another peak is detected. It is registered at 4.5 m height, whose value is lower than the peak located at 1.5 m. This is due to blast and deflagration generated by this kind of weapon.

We have also observed that the most pronounced structural movements are recorded at 1.5 m height. The time the structure needs to absorb and completely attenuate this movement is also maximum at this point. Another aspect also observed, and predictable, is that the structural movements are greater in the Z axis since it measures the transverse movements of the wall instead of the longitudinal movements along the length and width of the facade. Finally, we should denote that the highest vibration indexes are also registered around 1.5 m, which corresponds to the area of the greatest sound pressure level registered on the facade.

**Figure 24:**
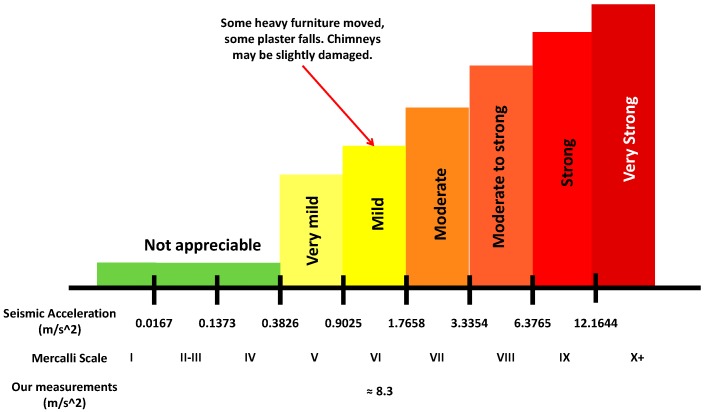
Mercalli scale and its damages.

Although we should not compare the structural movements produced by pyrotechnics and captured at the level of the façade with those generated by a natural earthquake, we can perform a small comparison in relation to the damages observed in both cases. To do this, we will use the Mercalli scale [29]. The Mercalli scale quantifies the effects of an earthquake on the Earth’s surface, humans, objects of nature, and man-made structures on a scale from I (not felt) to XII (total destruction). Figure 24 summarizes the ranges considered by this scale.

As we have seen in our results, the biggest vibration has been detected in the Z axis in which case we have obtained amplitudes of 133 dB (which is equivalent to 8.341 m/s^2^) for accelerometer 1. These values would imply a seismic intensity of grade IX in the Mercalli scale. 

**Figure 25:**
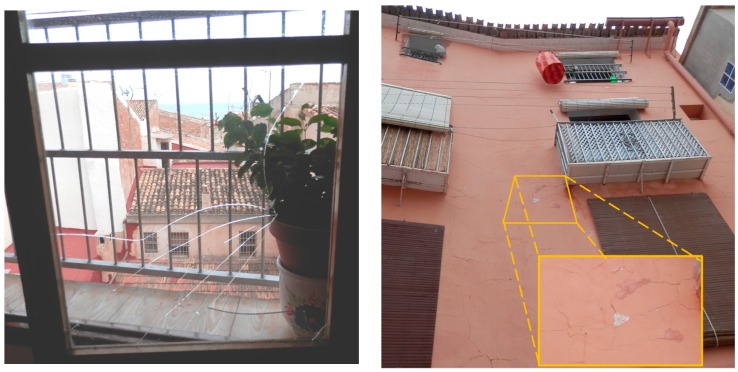
Some damages detected after tests.

Obviously, these damages are mitigated because the effect and vibrations are only registered at the facade level. However, if we consider the damages generated, we would be a seismic intensity of grade V-VI in the Mercalli scale. As Figure 25 shows, after performing our tests some damages have been detected in elements such as windows and plastering of the facades. Finally, we can conclude this section by affirming that the use of these weapons in a scenario like the one used in this tests can generate some structural damage, especially in elements like windows and plastering of facades. Consequently, the results of this study can help the organizers of these festivals in terms of security and damage prevention.

## 5. Conclusions

During the summer months, many European countries celebrate hundreds of festivals that commemorate historical events. Along the Spanish Mediterranean coast, the population often replicates ancient battles which involve plenty of shots of firearms and pyrotechnics along the historical center of the cities. These battles reproduce the events when Arab troops tried to conquer the Spanish land and how the Christian troops repelled these attacks. As a result of our history and all these facts, nowadays, we have many buildings like fortresses, watchtowers, churches and historic facades, which must be preserved to keep our history. However, so far we have not checked whether the reproduction of such celebrations near these historic buildings can cause some kind of irreparable damage to these buildings.

Because of this need, in this paper, we have presented a study about the vibroacoustic impact that buildings may suffer by the presence of pyrotechnics like the weapons typically used in these celebrations. To carry out this study, we used an old house where several sound level meters and vibration sensors have been placed over the facade. We have performed two sets of measurements. In both cases, six harquebusiers simultaneously shot their weapons.

From the results, we have seen that the greatest acoustic impact is recorded at 1.5 m height, although a higher sound pressure level is also recorded at 4.5 m height. As shown in Figure 16, when a weapon is fired, some deflagrations and explosions are generated. While the deflagration can reach up to 5–6 m height (this is reason of the high value of sound pressure level at 4.5 m), the explosion is generated next to the weapon mouth. In addition a small deflagration of the remaining gunpowder is registered near the handle of the weapon. Consequently, a high sound pressure level value has been registered at 1.5–2 m. These sound pressure level values has a consequence on the facade and in our case, this effect is the registration of some structural movements and vibrations which also increase with the height.

Finally, we can conclude that the height of the building must be taken into account when the vibroacoustic impact is measured. Obviously, another important factor is the height of the explosions, deflagrations and physical features of weapons. The results obtained demonstrate that the vibroacoustic effect on facade claddings in a bad state is high and it is capable of causing detachment, and human injuries (as Figure 22 shows). During the inventory of the damages caused, there were several broken windows, so these tests have served to determine the extent of the damage. The determination of human injuries, rather than the built heritage, will be decisive for limiting these traditional celebrations in historic centers of cities and towns.

These tests were carried out in old buildings but in the future, we would like to perform them on old architectural elements such as historical facades and watchtowers. In addition, we would like to implement a sensor network [30] able to take measurements for long periods using distributed database management techniques [31,32]. Moreover, we have in mind to create alarms [33] in cases where the building suffers some type of micro-vibration which can deteriorate the building structure somehow, when it has had a stressful event as the one reproduced in this work. Finally, we would like to test how these buildings can be affected by the explosions of aerial fireworks and compare these results with those obtained in the current paper.

## Figures and Tables

**Table 1:** Physical properties of black gunpowder.

Parameter	Pressure (P)	Kinetic Energy (F)	Volume (V)	Amount of Heat (Q)	Temperature (T)
Black Gunpowder	2970 kg/cm^2^	210 × 10^3^ kgm/kg	330 L	500 kcal/kg	2100 °C

**Table 2:** Features of weapons used during these tests.

Shooter	Kind of Weapon	Brand	Caliber (mm.)	Barrel Length (cm.)
1	Muzzle-loading Harquebus	BOPE	62	50
2	Blunderbuss	ARDESA	62	50
3	Muzzle-loading Harquebus	BOPE	28	41
4	Muzzle-loading Harquebus	GIL	28	40
5	Blunderbuss	BOPE	60	50
6	Blunderbuss	ARDESA	62	50
